# Developing an explainable machine learning model to predict false-negative citrin deficiency cases in newborn screening

**DOI:** 10.1186/s13023-025-04045-z

**Published:** 2025-10-08

**Authors:** Peiyao Wang, Haomin Li, Xinjie Yang, Lingwei Hu, Yuhe Chen, Ziyan Cen, Pingping Ge, Qimin He, Benqing Wu, Xinwen Huang

**Affiliations:** 1 Department of Genetics and Metabolism, Children’s Hospital of Zhejiang University School of Medicine, National Clinical Research Center for Child Health, No. 3333 Binsheng Road, Binjiang District, Hangzhou City, 310052 Zhejiang Province China; 2 Clinical Data Center, the Children’s Hospital, Zhejiang University School of Medicine, National Clinical Research Center for Child Health, Hangzhou, China; 3 School of Geography Science and Geomatics Engineering, Suzhou University of Science and Technology, Suzhou, 215009 China; 4 Children’s Medical Center, Shenzhen Guangming District People’s Hospital, Guangdong, 518106 China

**Keywords:** Citrin deficiency, Machine learning, Explainable AI, False-negative, Newborn screening

## Abstract

**Background:**

Neonatal Intrahepatic Cholestasis caused by Citrin Deficiency (NICCD) is an autosomal recessive disorder affecting the urea cycle and energy metabolism. Newborn screening (NBS) usually relies on elevated citrulline, but some patients have normal citrulline, resulting in false negatives and delayed diagnosis. This study develops an explainable machine learning (ML) model to predict false-negative NICCD cases during NBS.

**Methods:**

Data from 53 false-negative NICCD patients and 212 controls, collected retrospectively between 2011 and 2024, were analyzed. The dataset was split into a training set (70%) and a test set (30%). External validation involved 48 participants from distinct time periods. Key predictors were identified using variable importance in projection (VIP > 1) and Lasso regression. Six ML models were trained for evaluation: Logistic Regression, Random Forest, Light Gradient Boosting Machine, Extreme Gradient Boosting (XGBoost), K-Nearest Neighbor, and Support Vector Machines. Performance was evaluated using the area under the receiver operating characteristic curve (AUC) and F1 score. Shapley Additive exPlanations (SHAP) was applied to determine the importance of features and interpret the models.

**Results:**

Birth weight, citrulline, glycine, phenylalanine, ornithine, arginine, proline, succinylacetone, and C10:2 were selected as predictive features. Among the ML models, XGBoost demonstrated the most robust and consistent performance, achieving AUCs of 0.971(95%CI: 0.959–0.979), 0.968, and 0.977, and F1 scores of 0.786(95% CI: 0.744–0.820), 0.828, and 0.833 in the training, test, and external validation sets, respectively. SHAP analysis showed that the most important features are citrulline, glycine, phenylalanine, succinylacetone, birth weight, and ornithine. Feature pairs such as citrulline-phenylalanine, citrulline-glycine, succinylacetone-birth weight, and ornithine-glycine showed varying interactions. SHAP force plots, decision plots, and waterfall plots provided insightful patient-level interpretations. Finally, we built a network calculator for the prediction of false-negative NICCD cases (https://myapp123.shinyapps.io/my_shiny_app/).

**Conclusion:**

An interpretable machine learning model utilizing metabolite and demographic data enhances the detection of false-negative NICCD cases, facilitates early identification and intervention, and ultimately improves the overall effectiveness of the newborn screening system.

**Supplementary Information:**

The online version contains supplementary material available at 10.1186/s13023-025-04045-z.

## Introduction

Citrin deficiency (CD) is a mitochondrial disease caused by mutations in *SLC25A13*, leading to a defective urea cycle with three age-related manifestations: neonatal intrahepatic cholestasis caused by CD (NICCD), post-NICCD, including failure to thrive and dyslipidemia caused by citrin deficiency, and adolescent and adult citrin deficiency (AACD). Citrin functions as an essential carrier protein of the malate–aspartate shuttle (MAS), working in conjunction with the mitochondrial oxoglutarate carrier (OGC). This system enables oxidation of NADH in the cytosol and reduction of NAD⁺ in the mitochondrial matrix [1]. Loss of citrin function disrupts this balance, thereby impairing multiple metabolic pathways, including glycolysis and gluconeogenesis, de novo lipogenesis and beta-oxidation, and the tricarboxylic acid cycle [2]. In addition to maintaining redox homeostasis, citrin is also critical for exporting aspartate into the cytosol to sustain urea cycle flux. In CD, impaired aspartate export compromises urea cycle activity, contributing to the accumulation of toxic ammonia and the substrate citrulline (Cit) [3].

Based on these pathophysiological mechanisms, diagnosis of CD during newborn screening (NBS) typically relies on tandem mass spectrometry (MS/MS) to detect elevated Cit levels. However, some patients with delayed Cit elevation are missed by NBS (false negatives). These patients may later develop symptoms, and their diagnosis then relies on healthcare professionals recognizing the clinical features of NICCD [4]. Using Cit alone as an NBS marker has shown a sensitivity of only 43% and a positive predictive value of 7.7% [5], highlighting the need for more reliable biomarkers to improve early detection.

Given these shortcomings, several alternative strategies have been proposed to improve NICCD detection. One study proposed incorporating the Cit to total amino acids ratio (Cit/tAA) to enhance sensitivity and reduce missed diagnoses [6]. Another approach introduced second-tier molecular testing for SLC25A13 variants when Cit is above 20 umol/L to identify cases overlooked by MS/MS [7]. Furthermore, large-scale programs have combined MS/MS with high-throughput iPLEX genotyping to detect SLC25A13 mutations in newborns with borderline Cit levels [8]. Our previous study established a predictive formula incorporating Cit and selected amino acids and acylcarnitines to improve the detection of missed NICCD cases [9]. Other studies have also developed metabolite-based scoring systems to enhance the accuracy of MS/MS screening [10]. However, these methods still face limitations regarding reliance on fixed biochemical thresholds or predefined mutation panels, which may not fully capture the heterogeneity of NICCD metabolic profiles across different populations. Furthermore, some of these strategies either increase false positive rates or lead to additional testing costs or steps.

Machine learning (ML) has recently demonstrated strong performance in clinical prediction tasks [11]. Unlike conventional methods, ML can handle multiple features simultaneously and capture complex patterns among them, making it increasingly valuable in clinical research where outcomes often depend on interactions between diverse factors [12]. In the context of metabolic disease detection, previous studies have demonstrated the potential of ML [13]. For example, Haomin Li et al. successfully applied ML models to identify 11 inborn errors of metabolism using GC-MS urinary metabolomic data [14], supporting the feasibility of ML for rare disease screening. Despite these advancements, ML models are often limited by their lack of interpretability, creating a gap between model performance and clinical acceptance [15]. This tension between complexity and interpretability has limited the broader adoption of AI in healthcare. SHapley Additive exPlanations (SHAP) is a widely used method to address this issue by providing intuitive explanations for model predictions [16]. SHAP quantifies the contribution of each feature to individual predictions, helping to mitigate the “black box” nature of ML models.

Building on this foundation, our study aimed to address the persistent challenge of identifying false-negative NICCD cases with normal Cit levels during NBS - a well-known blind spot in current screening protocols - and to evaluate the clinical utility of interpretable ML models.

## Methods

### Study population

A total of 132 infants were admitted to the Children’s Hospital, Zhejiang University School of Medicine, between January 2011 and June 2024. These patients, who were typically diagnosed at 2–3 months of age, presented with jaundice, prolonged cholestasis, and failure to thrive, along with biochemical abnormalities (including citrullinemia, elevated bilirubin, and increased liver enzymes) in combination with pathogenic SLC25A13 variants, and were clinically diagnosed with later-onset NICCD. For these patients, retrospective review of dried blood spots (DBS) collected during newborn screening (NBS) was performed. Patients with unavailable or unclear DBS citrulline data were excluded (*n* = 79). Among the remaining 53 patients, all had normal citrulline levels, indicating false-negative NBS results. These patients were therefore classified as false-negative NICCD. We also included 66 true-positive NICCD cases identified through abnormal NBS (DBS citrulline > 37 µmol/L at 3–7 days after birth) and were confirmed by genetic testing.

Controls were consecutively enrolled from children attending routine physical examinations at the Department of Child Health Care during the same period. Among 265 children older than 6 months with retrievable NBS DBS records showing normal results, those with a history of abnormal liver function (*n* = 25) or metabolic abnormalities (*n* = 18), a family history of inherited metabolic disease (*n* = 4), or parental refusal (*n* = 6) were excluded, leaving 212 controls for the final analysis. The same inclusion and exclusion criteria were applied to the external validation cohort, which consisted of an independent set of 12 false-negative NICCD patients and 36 controls with NBS DBS samples collected between July 2024 and June 2025 at the same institution. Both case and control NBS DBS data were subsequently used for model construction, testing, and validation. This study was approved by the Ethics Committee of Children’s Hospital, Zhejiang University School of Medicine (reference number: 2021-IRB-292).

### Amino acids and acylcarnitines analysis in NBS

After informed consent was obtained from the parents of newborns, DBS cards were prepared in each maternity institution as part of a routine public health program. According to the standardized newborn screening protocol, blood samples were collected by heel-prick procedure at 3–7 days after birth and after adequate breastfeeding. The blood spots were dried at room temperature for 2–3 h before being sent to the Zhejiang Neonatal Disease Screening Center, one of China’s largest centers for genetic metabolic disorders.

Amino acids and acylcarnitines (including free carnitine and short-, medium-, and long-chain acylcarnitines) were measured using the NeoBase™ non-derivatized MS/MS Kit (PerkinElmer, Finland) on tandem mass spectrometry (MS/MS). Briefly, DBS punches were extracted with 100 µL of working solution containing stable isotope-labeled internal standards, shaken, and incubated at 45 °C for 45 min. After centrifugation and transfer, an aliquot of the supernatant was injected into the MS/MS system for analysis. Calibration was achieved using the stable isotope-labeled internal standards included in the kit. Three levels of internal controls (blank, low, and high) were applied to monitor assay performance. In addition, the laboratory participates in external quality assessment programs organized by the National Center for Clinical Laboratories (NCCL, China) and the Centers for Disease Control and Prevention (CDC, USA) to ensure accuracy and inter-laboratory comparability.

### Machine learning models

In this study, MS/MS-quantified metabolites (11 amino acids and 29 acylcarnitines) and demographic features (gender, birth weight, and gestational weeks) were available as potential ML input features. The dataset was randomly split into a 70% training set (*n* = 186) and a 30% test set (*n* = 79). In addition, a 10-fold cross-validation procedure was performed 10 times on the training data to assess model stability. Complicated high-dimensional data tends to affect the performance of ML algorithms; therefore, to minimize the risk of overfitting, feature selection using two methods in the training set was conducted before model construction. We employed Orthogonal Partial Least Squares Discriminant Analysis (OPLS-DA) to identify variables with variable importance in projection (VIP) scores greater than 1, and LASSO (Least Absolute Shrinkage and Selection Operator) regression, which can effectively reduce dimensionality by shrinking less important feature coefficients to zero. The intersection of variables identified by both methods was a candidate biomarker for NICCD prediction.

After the variable selection was completed, a total of six ML methods were used for model construction based on the training set: Logistic Regression (LR), Random Forest (RF), Extreme Gradient Boosting (XGBoost), Support Vector Machines (SVM), K-Nearest Neighbors (KNN), and Light Gradient Boosting Machine (LightGBM). Each model was chosen for its unique advantages; LR provides interpretability and handles linear data well, RF is known for its robustness and versatility [17], and XGBoost and LightGBM excel in prediction accuracy [18] and large-scale data processing [19], respectively. SVM [20] is effective for binary classification, and KNN performs well in transparent and easy-to-implement tasks [21]. The bootstrap method was implemented with 1000 replications to derive the confidence intervals (CI) of the area under the receiver operating characteristic curve (AUC), sensitivity, specificity, precision, and F1 score. The AUC and F1 scores were the major indices of the model performance comparison [22, 23]. Detailed parameter settings for all models are provided in the Supplementary Material (Tables S1–S6).

The final model with the best efficacy was elucidated using the SHapley Additive exPlanations (SHAP) algorithm, which quantifies each variable’s contribution to model predictions through attribution values (SHAP values). This approach enables both cohort-level and individual patient-level model interpretation [24]. We utilized multiple SHAP-based visualization techniques to comprehensively understand feature impacts and interactions. Key features were ranked according to their mean absolute SHAP values in the feature importance plot, while the summary plot demonstrated how feature values impacted model predictions and their direction. Dependence plots visualize the relationship between feature values and their SHAP contributions. For individual predictions, force plots, decision plots, and waterfall plots illustrated how specific feature combinations influenced the model’s decision pathway.

### Statistical analyses

**Fig. 1:**
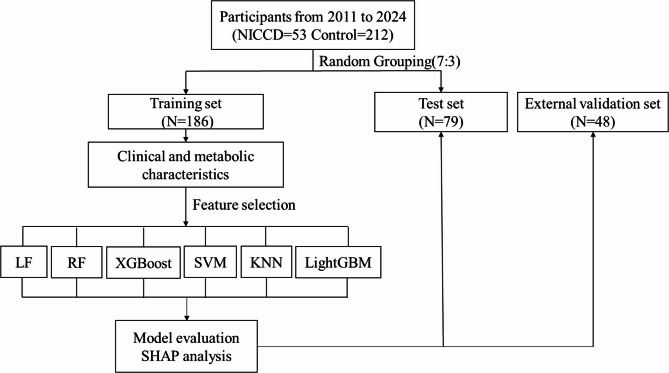
Flowchart summary of our methodology. LR, logistic regression; RF, random forest; XGBoost, extreme gradient boosting; SVM, support vector machine; K-NN, k-nearest neighbor; LightGBM, light gradient boosting machine

Continuous data are presented as mean and standard deviation or median and interquartile range (IQR), and categorical data are presented as percentages. Categorical variables were compared using a chi-squared test, while continuous variables were analyzed using an independent-samples t-test. Statistical significance was set at *P* < 0.05 (two-sided). All statistical analyses in this study were performed in R software R4.4.2 and Python 3.12.7 environments. The general overview flowchart of the study is presented in Fig. 1.

## Results

### Model biomarker selection

**Fig. 2:**
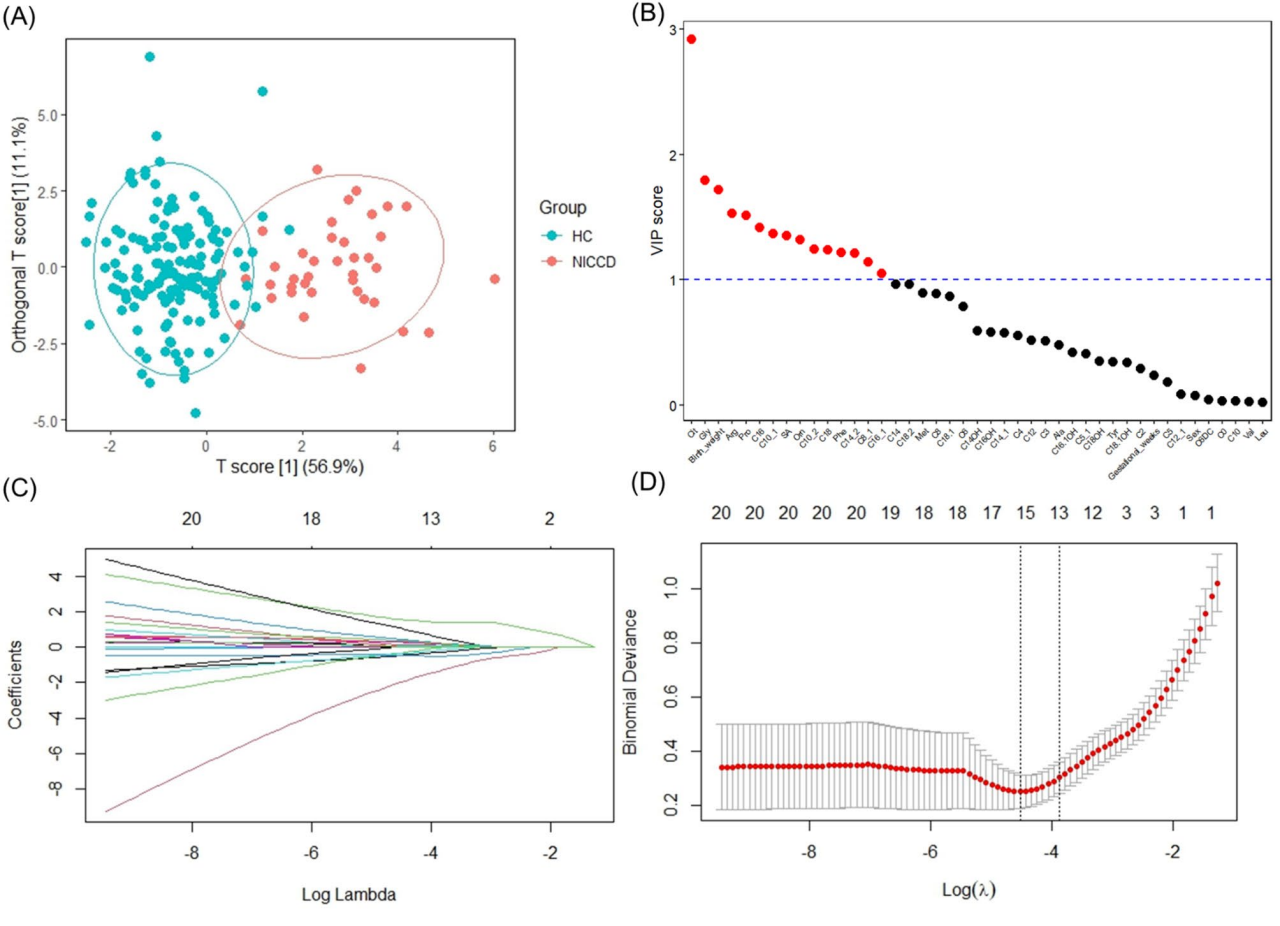
Identification of key variables distinguishing false negative NICCD patients and healthy controls using VIP and LASSO. **A** OPLS-DA score plot showing separation between the two groups. **B** Fifteen variables were selected based on their VIP scores > 1. **C** LASSO coefficient path plot. Coefficient changes as regularization strength increases, with more features eliminated as λ increases from right to left. **D** LASSO cross-validation error plot. The minimum criteria and the 1-standard error criteria were used to draw the dotted vertical lines at the optimal values of variables

To identify critical biomarkers for false-negative NICCD cases, we performed OPLS-DA to distinguish false-negative NICCD patients from healthy controls (HC). The score plot showed significant discrimination between the NICCD and HC groups (Fig. 2A). Fifteen variables with VIP scores > 1 were identified as core contributors, including Cit, glycine (Gly), birth weight (BW), arginine (Arg), proline (Pro), C16, C10:1, succinylacetone (SA), ornithine (Orn), C10:2, C18, phenylalanine (Phe), C14:2, C8:1, and C16:1 (Fig. 2B). Subsequently, Lasso regression was employed for feature selection (Fig. 2C). Using the lambda.1se criterion for model simplification (Fig. 2D), thirteen variables were selected: BW, gestational weeks, Cit, Phe, Arg, Orn, Gly, valine, Pro, C0, C8, SA, C10:2. The intersection of these two methods yielded nine candidate biomarkers for NICCD prediction: birth weight, Cit, Phe, Arg, Orn, Gly, Pro, SA, and C10:2.

### Baseline characteristics and biomarker profiles

The baseline characteristics and biomarker profiles of the cohort are summarized in Table 1. False-negative NICCD patients exhibited significantly lower BW compared to healthy controls (*P* < 0.001), while no significant differences were found in sex distribution or gestational age between the two groups (*P* > 0.05). Although all metabolic and clinical biomarkers fell within normal reference ranges, significant differences were still observed between false-negative NICCD cases and controls. Specifically, the levels of Cit, Arg, Orn, Pro, and SA were elevated in the false-negative NICCD cases, whereas Phe and Gly were lower compared to the control group. In true-positive NICCD cases, a similar pattern was observed, with elevated Cit, Arg, Orn, Pro, SA, C10:2, and Phe, and decreased Gly compared with controls (Table S7).

**Table 1:** Basic information, metabolic profiles in NBS, and clinical features at onset

	Training and test set			External validation set		
	NICCD(*N* = 53)	Controls(*N* = 212)	*P*	NICCD(*N* = 12)	Controls(*N* = 36)	*P*
Basic information						
Sex (n, %)			0.54			0.51
Male	25(47.17)	110(51.89)		5(41.67)	19(52.78)	
Female	28(52.83)	102(48.11)		7(57.33)	17(47.22)	
Birth Weight(g)	2865.57 ± 406.39	3274.36 ± 353.55	< 0.001	2906.67 ± 270.49	3203.06 ± 741.69	< 0.05
Gestational age (n, %)			0.44			0.73
< 28 weeks	0	0		0	0	
28-37weeks	2(3.77)	14(6.60)		1(8.33)	2(5.56)	
37-42weeks	51(96.22)	198(93.40)		11(91.67)	34(94.44)	
>42 weeks	0	0		0	0	
Metabolic profiles of DBS samples in NBS						
Cit (µmol/L)	25.50(19.82,30.03)	13.59(11.80,16.89)	< 0.001	24.79(19.18,28.13)	13.91(11.87,16.15)	< 0.001
Phe (µmol/L)	49.60(43.92,55.60)	59.00(52.52,66.62)	< 0.001	53.05(38.89,59.27)	57.41(53.09,71.94)	< 0.05
Arg (µmol/L)	10.58(4.98,16.06)	5.33(2.93,8.61)	< 0.001	11.91(2.41,19.38)	5.52(2.81,9.37)	0.15
Orn (µmol/L)	149.62(129.60,182.92)	126.64(100.88,150.54)	< 0.001	140.05(114.92,157.44)	121.35(97.43,155.38)	0.53
Gly (µmol/L)	390.39(341.44,478.79)	535.49(453.84,634.52)	< 0.001	389.30(320.09,415.34)	571.97(502.42,710.68)	< 0.001
Pro (µmol/L)	240.23(196.16,297.43)	200.57(175.78,241.13)	< 0.001	239.28(192.31,271.78)	196.41(157.07,223.84)	< 0.05
SA (µmol/L)	0.80(0.68,0.95)	0.68(0.50,0.89)	< 0.001	0.88(0.58.1.25)	0.51(0.42,0.81)	< 0.01
C10:2 (µmol/L)	0.01(0.01,0.02)	0.01(0.01,0.01)	< 0.001	0.02(0.01,0.02)	0.01(0.01,0.01)	< 0.05
Clinical features at onset						
Onset age(d)	74(59,105)	-	-	71(55,95.5)	-	-
TP (g/L)	45.6(42.55,50.65)	-	-	42.35(39.48,50.95)	-	-
TBil (µmol/L)	142.3 (89.15,181.75)	-	-	155.95(70.9,181.05)	-	-
DBil (µmol/L)	53.5 (37.75,69.75)	-	-	79.75(31.28,73.6)	-	-
GGT (U/L)	197(117.5,281.5)	-	-	197.5(131.5,230.25)		
TBA (µmol/L)	236 (161.85,301.1)	-	-	264.05(222,295.13)	-	-
ALT (U/L)	40.5 (29.75,59)	-	-	49.5(32.5,67)	-	-
AST (U/L)	123 (79.5,185)	-	-	126.5(83.5,154)	-	-
TG (mmol/L)	1.20(1.52, 1.97)	-	-	1.76(1.42,1.87)	-	-
TC (mmol/L)	3.46(4.66, 5.60)	-	-	4.15(3.91,4.62)	-	-
PT (s)	15.35 (14.13,17.3)	-	-	15.2(13.2,19.15)		
Lac (mmol/L)	3.6(2.55,5.45)	-	-	3.65(2.92,4.17)		
Hb(g/L)	102.5(93, 109.25)	-	-	103.5(98.25,111.25)		

Data are median (Q1, Q3) or mean ± standard deviation

Normal range: Cit: 7.9–37µmol/L; Phe: 23.3–100µmol/L; Arg:2.54-50µmol/L; Orn: 52.09-323.22µmol/L; Gly:246.57–1283µmol/L; Pro: 97.2-401.5µmol/L; SA:0.16–2.58µmol/L; C10:2: 0.01–0.08µmol/L; TP: 57–80 g/L; TBil: 5–21µmol/L; Dbil: 0-5.1µmol/L; GGT: 8-57U/L; TBA: 0–12µmol/L; ALT: 5-50U/L; AST: 15-60U/L; TG: <1.7mmol/L; TC: 3-5.7mmol/L; PT: 9–14 s; Lac: 0.5-1.6mmol/L; Hb :110–155 g/L

The false-negative NICCD patients presented with clinical symptoms at a median age of 74 days in internal cases and 71 days in external cases. At disease onset, all patients exhibited markedly elevated levels of total bile acids (TBA), total bilirubin (TBil), direct bilirubin (DBil), and γ-glutamyl transferase (GGT). Liver injury was evident, with elevated levels of alanine aminotransferase (ALT) and aspartate aminotransferase (AST), the latter showing more pronounced elevation. Coagulation dysfunction was indicated by a prolonged prothrombin time (PT). Fewer than half of the patients demonstrated dyslipidemia. Additionally, increased lactate levels and decreased hemoglobin (Hb) concentrations were observed.

### Model evaluation and comparison

**Fig. 3:**
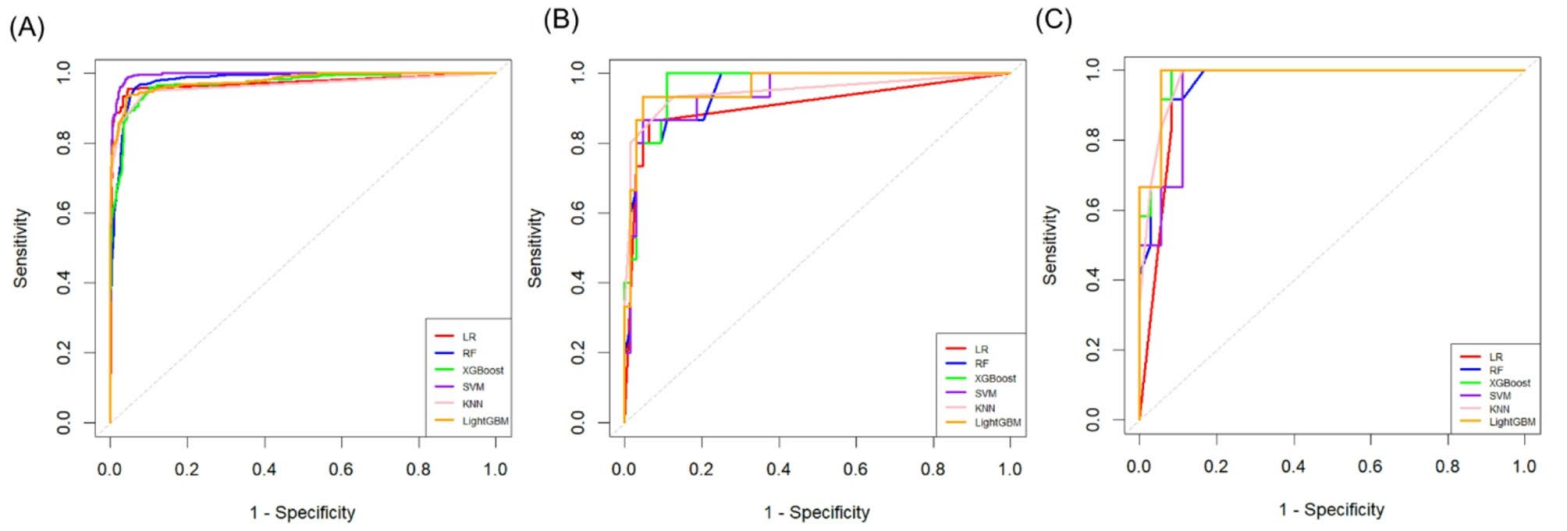
Receiver operating characteristic curves showing the predictive performance of machine learning models for NICCD detection in **A** training, **B** test, and **C** external validation set

Figure 3 presents the performance evaluation of six ML models (LR, RF, XGBoost, SVM, KNN, and LightGBM), including their ROC curves and key metrics. The AUC and F1 score were chosen as primary evaluation criteria, with the F1 score being particularly informative for handling class imbalance, as it harmonizes precision and recall. In the training set, SVM achieved the best performance with an AUC of 0.996 (95% CI: 0.993–0.998) and an F1 score of 0.913 (0.891–0.933) (Table 2). In the test set, however, XGBoost demonstrated the most balanced performance across multiple metrics, with an AUC of 0.968 and an F1 score of 0.828 (Table 3). Importantly, performance on the external validation set was summarized in Table 4, providing evidence of model generalizability. In this cohort, LightGBM and XGBoost achieved the two highest AUC values (0.981 and 0.977, respectively), while XGBoost yielded a higher F1 score (0.833 vs. 0.727 for LightGBM). Other models showed inferior or less stable performance in the external validation cohort. Taken together, XGBoost maintained comparably strong and consistent performance across test and external validation cohorts and was selected for subsequent SHAP-based interpretability analysis.

**Table 2:** Evaluation of model performance in the training set

	AUC	Accuracy	Sensitivity	Specificity	Precision	F1 Score
LR	0.971 (0.960–0.984)	0.959 (0.950–0.967)	0.884 (0.856–0.913)	0.978 (0.970–0.986)	0.927 (0.901–0.952)	0.893 (0.872–0.916)
RF	0.984 (0.978–0.988)	0.934 (0.923–0.944)	0.794 (0.749–0.836)	0.970 (0.961–0.978)	0.895 (0.864–0.924)	0.821 (0.791–0.850)
XGBoost	0.971 (0.959–0.979)	0.922 (0.908–0.934)	0.743 (0.702–0.786)	0.969 (0.958–0.977)	0.880 (0.838–0.909)	0.786 (0.744–0.820)
SVM	0.996 (0.993–0.998)	0.968 (0.960–0.975)	0.882 (0.846–0.906)	0.991 (0.984–0.995)	0.968 (0.950–0.981)	0.913 (0.891–0.933)
KNN	0.964 (0.950–0.978)	0.942 (0.932–0.951)	0.738 (0.697–0.782)	0.994 (0.991–0.999)	0.975 (0.947–0.988)	0.821 (0.784–0.853)
LightGBM	0.980 (0.973–0.989)	0.949 (0.940–0.959)	0.863 (0.828–0.902)	0.970 (0.962–0.977)	0.894 (0.864–0.918)	0.865 (0.836–0.890)

**Table 3:** Evaluation of the model performance in the test set

	AUC	Accuracy	Sensitivity	Specificity	Precision	F1 Score
LR	0.908	0.911	0.800	0.938	0.750	0.774
RF	0.952	0.924	0.733	0.969	0.846	0.786
XGBoost	0.968	0.937	0.800	0.969	0.857	0.828
SVM	0.946	0.924	0.800	0.953	0.800	0.800
KNN	0.948	0.937	0.733	0.984	0.917	0.815
LightGBM	0.964	0.924	0.733	0.969	0.846	0.786

**Table 4:** Evaluation of the model performance in the external validation set

	AUC	Accuracy	Sensitivity	Specificity	Precision	F1 Score
LR	0.951	0.917	0.917	0.917	0.786	0.846
RF	0.971	0.917	0.833	0.944	0.833	0.833
XGBoost	0.977	0.917	0.833	0.944	0.833	0.833
SVM	0.954	0.854	0.667	0.917	0.727	0.696
KNN	0.975	0.896	0.667	0.972	0.889	0.762
LightGBM	0.981	0.875	0.667	0.944	0.800	0.727

### Model interpretation

**Fig. 4:**
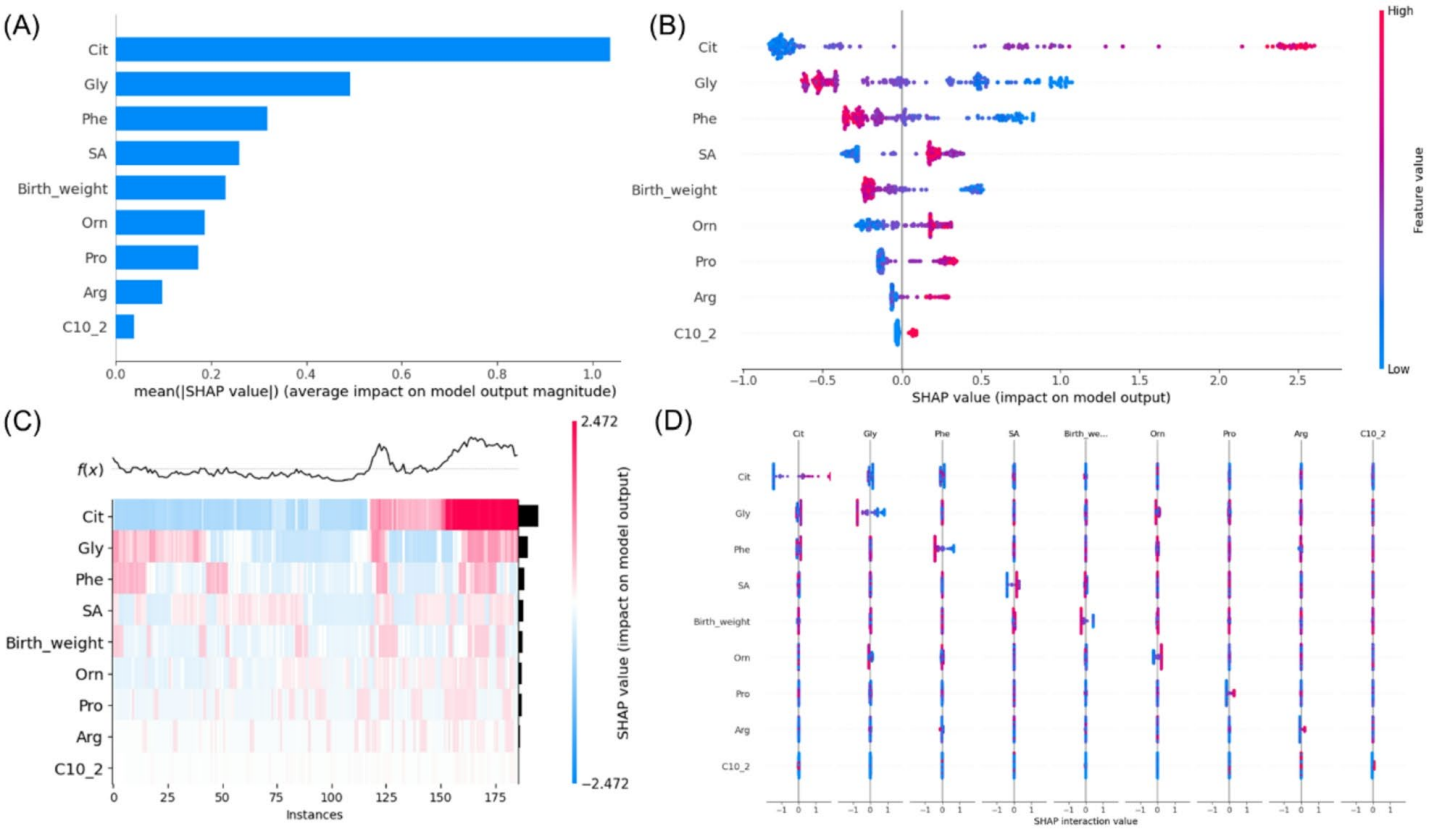
SHAP analysis of feature importance and interactions. **A** Ranking of feature importance based on mean absolute SHAP values. **B** SHAP summary plot showing feature impact on predictions, with colors indicating feature values (red = high, blue = low). **C** SHAP value distribution heatmap across instances, with colors showing feature contribution direction (blue = negative, red = positive) and f(x) displaying model output trend. **D** SHAP interaction plot displaying the strength of pairwise feature interactions, with wider distributions indicating stronger interactions

We performed a SHAP analysis to evaluate feature importance and their contributions to predictions in the XGBoost model (Fig. 4). Figure 4A displays the SHAP feature importance, with features ranked by their mean absolute SHAP values. The features were ranked in order of importance as follows: Cit, Gly, Phe, SA, BW, Orn, Pro, Arg, and C10:2. The SHAP summary plot (Fig. 4B) illustrates how each feature impacts model predictions. Higher SHAP values indicate a greater likelihood of NICCD diagnosis. For example, patients with elevated Cit are more likely to be diagnosed with NICCD compared to those with decreased levels. In addition, patients with decreased Gly or Phe are more likely to be diagnosed with NICCD than those with increased levels. The SHAP value distribution heatmap across all instances in the training dataset is shown in Fig. 4C. The SHAP interaction plot (Fig. 4D) displays both main effects (diagonal elements) and interaction effects (off-diagonal elements) between variables. The analysis demonstrated that variable interactions were primarily observed in the Cit-Phe, Cit-Gly, SA-birth weight, and Orn-Gly pairs, which showed effects on prediction outcomes.

**Fig. 5:**
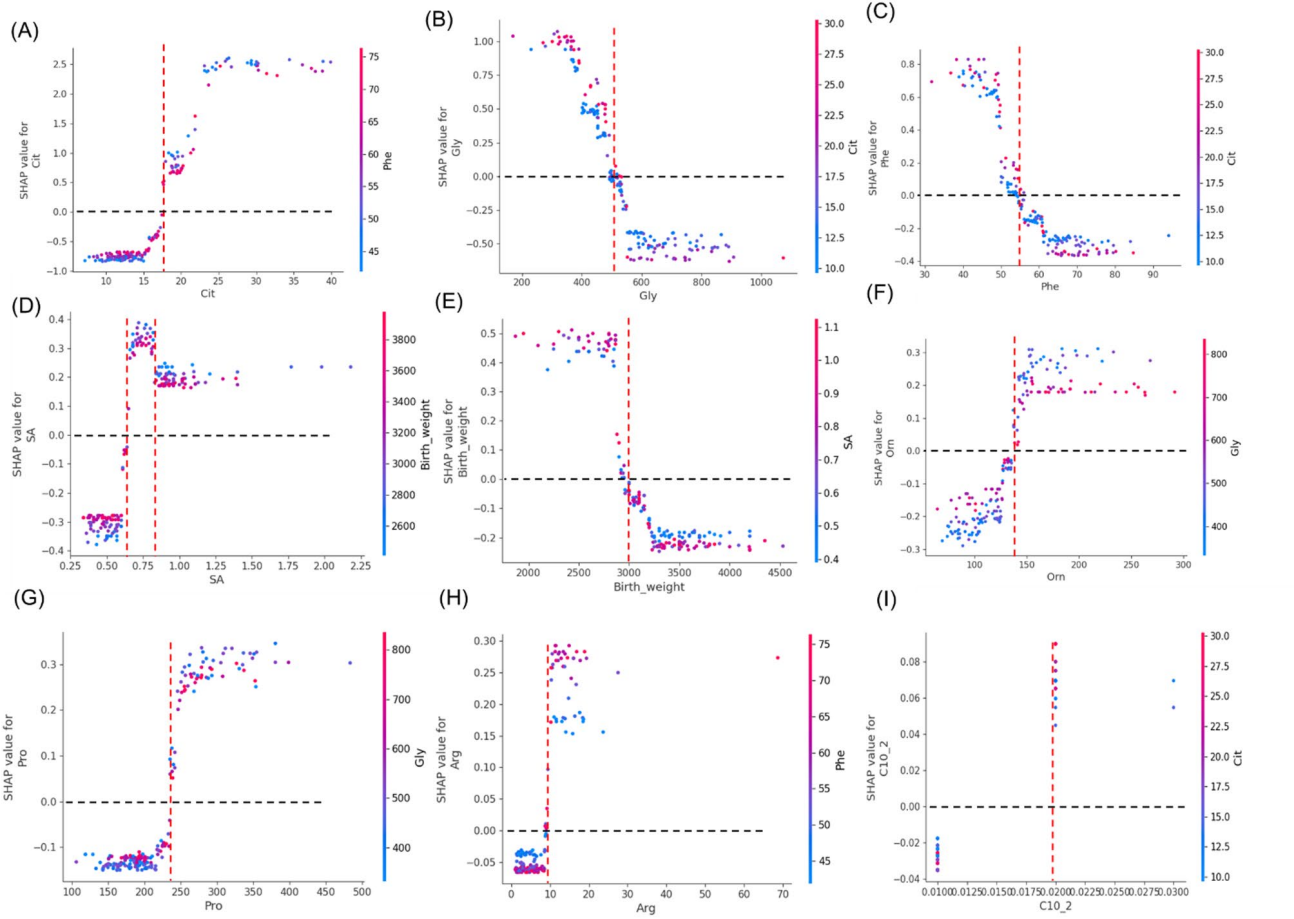
SHAP dependence plots of the top 6 variables. Each plot shows how feature values (x-axis) impact model predictions (SHAP values, y-axis). Point colors represent the concentration of the feature with the strongest interaction effect: **A** Cit vs. Phe, **B** Gly vs. Cit, **C** Phe vs. Cit, **D** SA vs. Birth weight, **E** Birth weight vs. SA, **F** Orn vs. Gly, **G** Pro vs. Gly, **H** Arg vs. Phe, **I** C10:2 VS Cit

Analysis of all 9 predictors showed that Cit displayed consistently negative SHAP values below 18, above which SHAP values increased dramatically with rising Cit concentrations. At higher Cit levels, lower Phe levels (blue) were associated with higher SHAP values (Fig. 5A). Phe levels below 55 µmol/L and Gly levels below 500 µmol/L corresponded to higher SHAP values, particularly when accompanied by elevated Cit (red) in the positive SHAP region (Fig. 5B and C). SA exhibited peak SHAP values at 0.7–0.8µmol/L, declining but remaining positive afterward, where lower birth weights demonstrated higher SHAP values (Fig. 5D). SHAP values were positive for birth weights under 3000 g but negative above this threshold. Higher SA values correlated with increased SHAP values for low birth weights, indicating higher NICCD risk (Fig. 5E). Orn levels showed a positive correlation with SHAP values until Orn reached around 150µmol/L, beyond which SHAP values stabilized, where lower Gly levels were associated with higher SHAP values (Fig. 5F). In addition, SHAP values became consistently positive when Pro exceeded approximately 250 µmol/L, Arg exceeded 10 µmol/L, and C10:2 exceeded approximately 0.02 µmol/L (Fig. 5G-I).

### Explanation of the machine learning model at the patient level

**Fig. 6:**
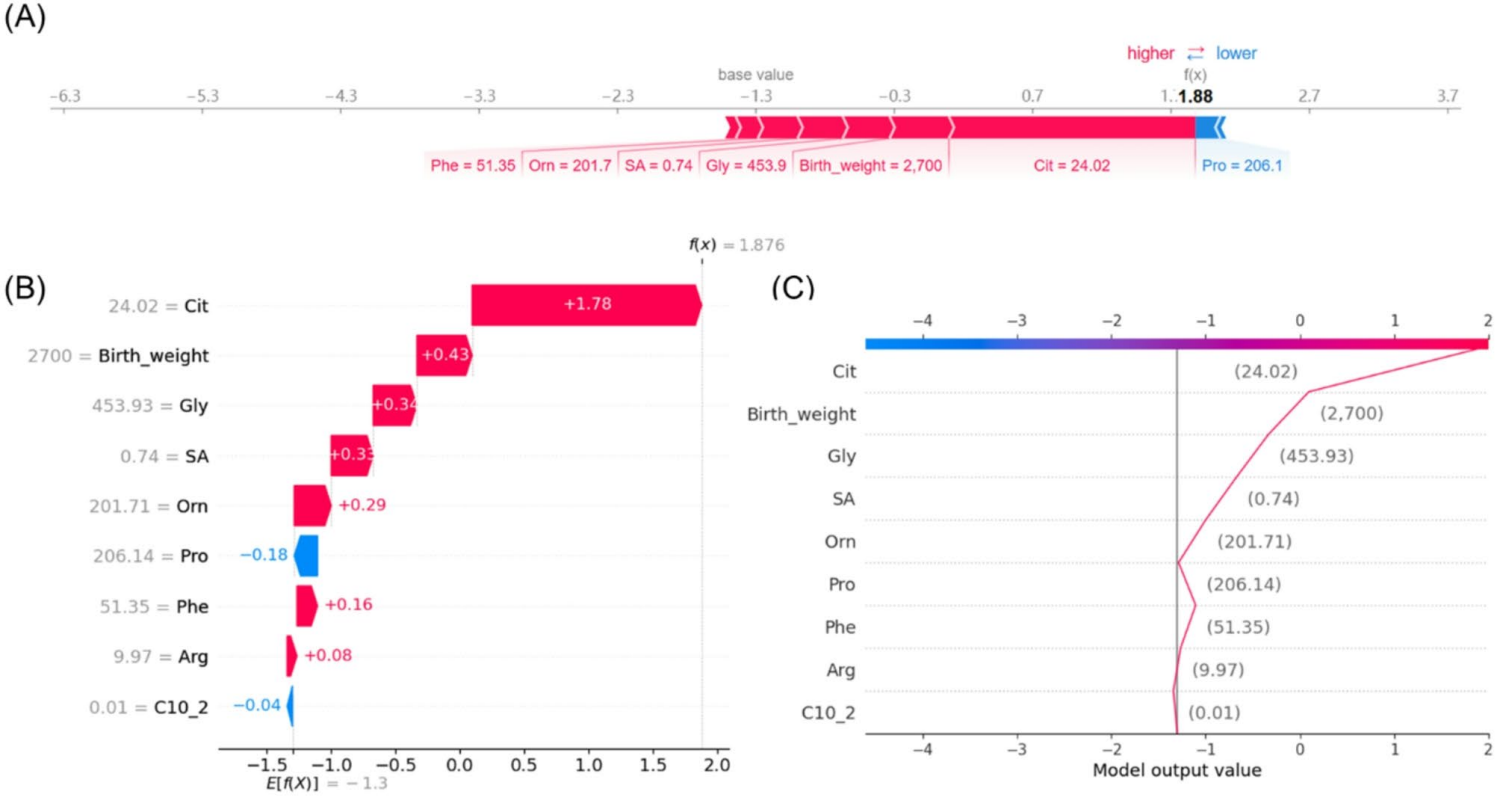
Prediction visualization for a false-negative NICCD patient. **A** Force plot. Red/blue indicating positive/negative impacts, and bar length representing contribution magnitude. **B** Waterfall plot. **C** Decision plot. The x-axis shows the model output value, while feature values are listed on the left side

**Fig. 7:**
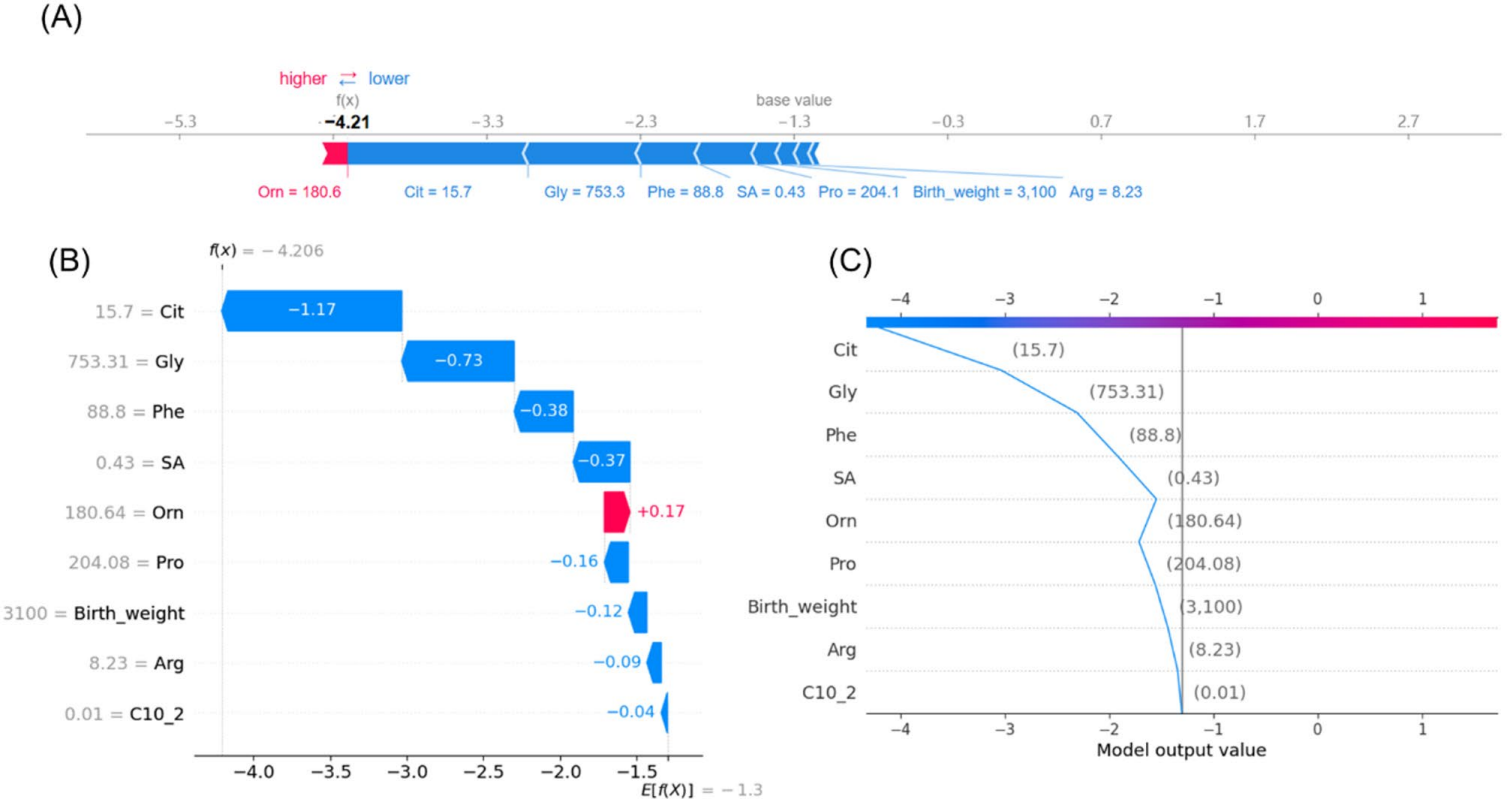
Prediction visualization for a true-negative healthy control. **A** Force plot. Red/blue indicating positive/negative impacts, and bar length representing contribution magnitude. **B** Waterfall plot. **C** Decision plot. The x-axis shows the model output value, while feature values are listed on the left side

**Fig. 8:**
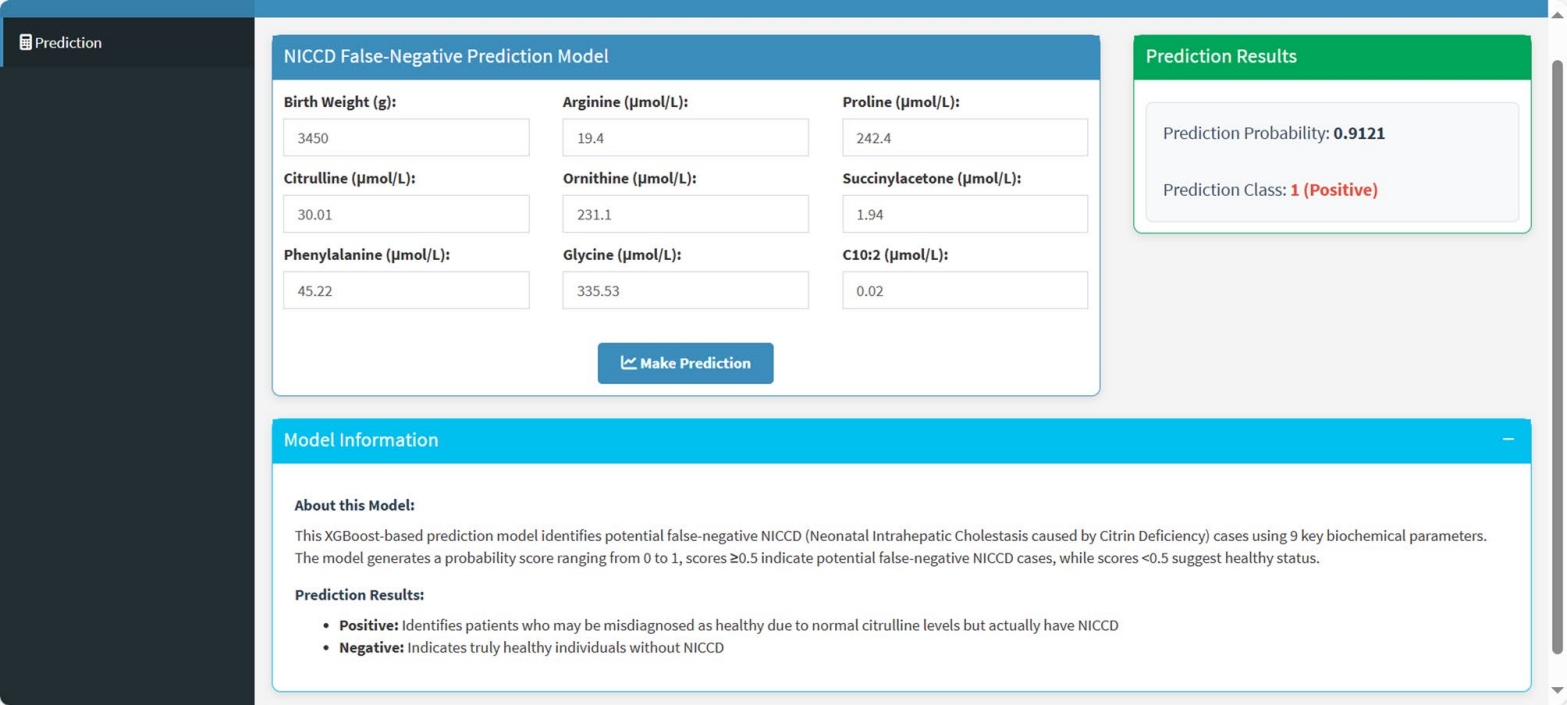
Network calculator predicting false-negative NICCD with normal Cit levels

To evaluate the contributions of features for individual patients using the XGBoost model, we applied the SHAP method to explain the individual predictions for two individuals. The color represents the contributions of each feature, with red being positive and blue being negative. The length of the color bar represents the contribution strength. For case A (false-negative), it yielded a prediction score of 1.88 (corresponding to 86.8% probability, through the sigmoid function [P(NICCD) = 1/(1 + e^(-f(x)))]), indicating a high likelihood of NICCD (Fig. 6); while for control B (control), it yielded a score of -4.21 (corresponding to 1.5% probability), suggesting a very low likelihood of NICCD diagnosis (Fig. 7). Finally, we developed a web-based calculator using a Shiny application to predict false-negative NICCD cases. As shown in Fig. 8, the functional cure prediction tool is available at the following website: https://myapp123.shinyapps.io/my_shiny_app/.

## Discussion

This is the first study to utilize machine learning models for screening and predicting NICCD patients based on nine readily accessible indicators from NBS (Cit, Gly, Phe, SA, BW, Orn, Pro, Arg, and C10:2). We evaluated six machine learning methods, among which the XGBoost model demonstrated superior performance. Furthermore, we employed SHAP analysis to interpret the XGBoost model, which provides rational explanations for both positive and negative factors influencing individual NICCD occurrence probability.

The diagnostic criteria of NICCD remain elusive due to its non-specific clinical manifestations at birth [25]. While traditional diagnostic approaches rely heavily on DBS Cit levels during NBS and genetic testing, an increasing number of researchers have reported false-negative cases with initially normal or borderline Cit levels that significantly elevate upon subsequent clinical manifestations, such as cholestasis. The mechanisms underlying the phenotypic variability during the neonatal period remain unclear, but one possibility is that blood sampling is being taken too early, before the metabolic profile has fully developed [26]. Additionally, the glycerol phosphate shuttle activity is an alternative mechanism for oxidizing NADH and generating energy, which may compensate for the loss of citrin function [27]. This pathway typically has lower activity in the human liver compared to other tissues. When this compensatory mechanism eventually becomes overwhelmed, clinical manifestations may emerge, including delayed onset of cholestasis in infancy, potentially explaining the variable timing and severity of symptom presentation in patients with the same genetic defects. Importantly, delayed identification typically correlates with poorer clinical outcomes, highlighting the need for improved screening strategies [28].

Metabolite concentrations in DBS are influenced by various factors, including nutritional intake, maternal factors, and seasonal fluctuations [29–31]. Considering this complexity, our study incorporated not only 40 metabolic markers from NBS but also crucial clinical variables such as sex, gestational age, and birth weight. We employed LASSO regression and OPLS-DA for feature selection, which identified 9 key variables most strongly associated with false-negative NICCD cases. While several of these markers (Cit, Phe, Orn, Gly, and Arg) align with biomarkers identified in previous studies, we also discovered novel predictive features - BW, Pro, SA, and C10:2 - that have not been previously emphasized in NICCD screening literature. These newly identified markers may provide important insights into the pathophysiological mechanisms underlying NICCD cases that escape conventional screening methods.

After conducting feature selection, we evaluated six machine learning algorithms (LR, RF, SVM, KNN, LightGBM, and XGBoost) through comprehensive cross-validation. Among these, the XGBoost model demonstrated superior performance. XGBoost, a supervised tree-based machine learning approach developed by Chen et al. in 2016, has consistently shown exceptional performance in classification and prediction tasks [18]. In the field of precision medicine, XGBoost has been successfully applied to various challenges, including chronic kidney disease diagnosis [32], intraoperative hypoxemia risk prediction [33], prediction of the development of acute kidney injury [34], and chronic obstructive pulmonary disease prediction [35]. However, to our knowledge, this is the first application of XGBoost for NICCD prediction. Citrin deficiency disrupts the transport of aspartate into the cytoplasm and glutamate into the mitochondria, resulting in the accumulation of intermediate products of the urea cycle and perturbation of multiple metabolic pathways. These metabolic disturbances create complex network patterns rather than simple linear relationships among biochemical parameters. XGBoost demonstrated superior predictive performance, which can be attributed to its exceptional ability to handle complex non-linear relationships between features, thus outperforming traditional linear models such as logistic regression [36].

Furthermore, SHAP value analysis revealed the contribution of various indicators to NICCD prediction and their complex interactions, providing new perspectives for understanding the metabolic characteristics of NICCD. Our study suggests that although Cit, Orn, and Arg are within the normal range, they are elevated compared to controls, while Gly is decreased, resembling the metabolic pattern seen in true-positive NICCD patients. SHAP analysis further showed that these metabolic changes improve the model’s ability to predict false-negative NICCD. Citrin deficiency reduces liver cytosolic aspartate, a key substrate of argininosuccinate synthetase (ASS) in the urea cycle, leading to the accumulation of citrulline (a direct substrate of ASS) and ornithine (an upstream substrate in the same cycle) [37]. Arginine, a downstream product of ASS in the urea cycle and primarily synthesized from citrulline in the kidneys and small intestine, was elevated because accumulated citrulline in NICCD accelerates this extrahepatic synthesis. Additionally, citrin participates in the aspartate-malate shuttle system, which is responsible for oxidizing cytosolic NADH produced by glycolysis and reducing NAD+ in the mitochondrial matrix. Dysfunctional citrin impairs hepatic glycolysis and reduces the efficiency of ATP formation. As glycolysis and lactate gluconeogenesis become impaired [38], the body shifts toward relying on gluconeogenic amino acids like glycine for compensatory glucose production, resulting in significantly decreased glycine levels.

In this study, we observed that approximately 35% of true-positive NICCD patients identified through NBS exhibited elevated Phe levels. By contrast, no such elevation was seen in the false-negative group, where Phe levels were even lower than those of controls. Consistent with our findings, previous research also reported that false-negative NICCD patients had slightly lower Phe levels than controls (46.9 vs. 48 µmol/L), although this difference was not statistically significant [6], possibly due to limited sample size (*n* = 12). This metabolic difference is likely explained by the fact that in true-positive patients, cholestasis and liver dysfunction impair aromatic amino acid metabolism, resulting in elevated Phe levels [39], whereas false-negative patients do not exhibit overt liver dysfunction at the time of NBS, possibly owing to compensatory biochemical and physiological mechanisms [40]. These findings suggest that lower Phe levels in the NBS could potentially serve as an early warning signal for false-negative NICCD.

SA is a byproduct of tyrosine catabolism, generated in the liver via fumarylacetoacetate accumulation and excreted in urine [41]. In our study, SA was identified by our model as a predictive variable, with SHAP analysis revealing a non-linear association of SA with false-negative NICCD. We speculate that this pattern might reflect subtle perturbations in hepatic tyrosine metabolism in NICCD cases, which could be captured by a machine learning model as a potential risk signal. This observation warrants further investigation in future studies. We also observed a strong interaction between higher SA and lower birth weight, with their combination increasing NICCD prediction probability. Impaired carbohydrate metabolism in CD reduces de novo lipogenesis [42], compromising third-trimester fetal myelination and fat deposition, resulting in low birth weight and height in affected infants [43, 44]. Previous studies recommend genetic testing for SLC25A13 variants in newborns with reduced height/weight and a score ≥ 4 on the score system to avoid missing CD diagnosis [10]. C10:2 carnitine was also identified as a discriminative variable, with slightly higher levels contributing positively to false-negative NICCD cases. The main regulator of fatty acid oxidation, peroxisome proliferator-activated receptor α (PPARα), has been reported to be downregulated in citrin deficiency [45]. This downregulation may suppress mitochondrial β-oxidation and potentially affect enzymes involved in unsaturated fatty acid oxidation, such as 2,4-dienoyl-CoA reductase, thereby contributing to the subtle difference in C10:2.

SHAP interaction analysis reveals that when Cit is high and Phe is low, the SHAP value increases, indicating that this combination significantly enhances the likelihood of predicting false-negative NICCD cases. Even if Cit levels do not reach the conventional diagnostic threshold for a significant increase, the combination with low Phe levels may still serve as an important predictor for NICCD. This explains why relying solely on Cit levels for screening may lead to false negatives. Similarly, when Gly is low and Cit is high, Orn is high and Gly is low, and SA is high and birth weight is low, these specific metabolite combinations also increase the likelihood of predicting false-negative NICCD cases. This indicates that metabolite combinations and their interactions have higher predictive effectiveness in NICCD screening.

Finally, we visualized the decision-making process of the XGBoost model using two representative samples (one false-negative and one control), which effectively revealed the inner logic of the “black box” model. This level of interpretability not only improves clinicians’ trust in AI-assisted screening tools but also enables physicians to communicate diagnostic reasoning to patients and families [46]. In addition, by using the Shinyapps platform, we integrated our prediction model into a user-friendly, web-based calculator primarily designed for clinicians. This interactive tool allows clinicians to input patient-specific variables and instantly obtain individualized risk predictions. It is simple to operate, requires no additional software, and provides rapid feedback that can be readily incorporated into daily clinical workflow, thereby improving clinicians’ efficiency. Being web-based, the calculator is accessible across different clinical settings, facilitating broader adoption.

Our findings have important clinical implications. The model serves as a useful supplement to conventional NBS by identifying false-negative cases with normal citrulline levels that would otherwise be missed, thereby enabling earlier recognition, timely treatment, and improved long-term outcomes for affected infants. For clinicians, it provides quantitative, evidence-based support that promotes more objective and consistent decision-making. Moreover, our results contribute to a better understanding of the pathophysiology of NICCD and related metabolic disorders, advancing rare disease research.

Despite the highlighted advantages of the proposed approach, there are several limitations worth mentioning. First, our model was developed in a single center, and the limited number of false-negative NICCD cases increases the risk of overfitting. Although penalization, cross-validation, and external validation were applied, residual overfitting cannot be fully excluded. Second, our results are based on a single MS/MS platform and may not be directly generalizable to other analytical methods or laboratories using different reference ranges. Taken together, these limitations highlight the need for future validation in larger, multi-center cohorts employing diverse methodologies to establish broader applicability. Finally, the model relies solely on blood tandem mass spectrometry data. Future improvements should integrate additional clinical risk factors, including seasonal variations, maternal factors, blood collection timing, and urinary organic acid profiles. This multidimensional approach would develop more robust and precise prediction models, improving the sensitivity-specificity balance and maximizing clinical utility.

## Conclusion

Our ML approach complements the mono-metabolite citrulline method by enhancing the detection of false-negative cases with normal citrulline levels. With the SHAP method, we achieve interpretable predictions and build transparent models. To support clinical adoption, we also developed an online calculator based on the optimized model. Collectively, these approaches enhance parental counseling, inform targeted therapeutic interventions, guide the refinement of NBS protocols, and facilitate broader clinical implementation.

## Supplementary Information

Below is the link to the electronic supplementary material.

Supplementary Material 1

## Data Availability

Some or all datasets generated during and/or analyzed during the current study are not publicly available regarding patient privacy and confidentiality but are available from the corresponding author upon reasonable request.

## Abbreviations

NICCD: Neonatal intrahepatic cholestasis from citrin deficiency
CD: Citrin deficiency
Cit: Citrulline
ML: Machine learning
SHAP: Shapley Additive exPlanations
LR: Logistic Regression
RF: Random Forest
XGBoost: Extreme Gradient Boosting
SVM: Support Vector Machines
KNN: K-Nearest Neighbor
LightGBM: Light Gradient Boosting Machine
Gly: Glycine
Arg: Arginine
Pro: Proline
SA: Succinylacetone
Orn: Ornithine
Phe: Phenylalanine: BW: birth weight
