# Supplementary material for: Developing an explainable machine learning model to predict false-negative citrin deficiency cases in newborn screening
Source: Orphanet J Rare Dis. 2025 Oct 8;20:507. doi: 10.1186/s13023-025-04045-z (PMC12505637; doi:10.1186/s13023-025-04045-z)
Supplement: Supplementary file 1 — Supplementary Material 1 [file 13023_2025_4045_MOESM1_ESM.docx]

**Table S1. Key parameters of the Logistic Regression**

| Parameter Name | Value | Description |
| --- | --- | --- |
| family | binomial | Distribution family for binary classification |
| link | logit | Link function: log(p/(1-p)) |
| method | IWLS | Iterative Weighted Least Squares fitting method |
| maxit | 25 | Maximum number of iterations for convergence |
| epsilon | 1e-8 | Convergence tolerance criterion |

**Table S2. Key parameters of the Random Forest**

| Parameter Name | Value | Description |
| --- | --- | --- |
| mtry | 3 | Number of variables randomly sampled as candidates at each split |
| ntree | 50 | Number of trees to grow |
| nodesize | 1 | Minimum size of terminal nodes |
| maxnodes | NULL | The maximum number of terminal nodes tree can have |
| sampsize | N | Size of sample to draw (N = training set size) |
| replace | TRUE | Should sampling of cases be done with or without replacement |

**Table S3. Key parameters of the XGBoost**

| Parameter Name | Value | Description |
| --- | --- | --- |
| nrounds | 150 | Number of boosting iterations (trees to build). |
| max_depth | 3 | Maximum depth of a tree; controls model complexity and potential overfitting. |
| eta | 0.03 | Learning rate, step size shrinkage is used in the update to prevent overfitting. |
| gamma | 1 | Minimum loss reduction required to make a further partition on a leaf node. |
| colsample_bytree | 0.6 | Fraction of features to randomly sample for each tree. |
| min_child_weight | 3 | Minimum sum of instance weight (hessian) needed in a child node. |
| subsample | 0.6 | Fraction of the training data to be randomly sampled for each tree. |
| lambda | 1 | L2 regularization term on weights (ridge regression). |
| alpha | 0.5 | L1 regularization term on weights (lasso regression). |

**Table S4. Key parameters of the SVM**

| Parameter Name | Value | Description |
| --- | --- | --- |
| kernel | radial | Radial basis function (RBF) kernel |
| sigma | 0.1 | Kernel parameter for RBF kernel |
| C | 10 | Cost parameter for regularization |
| type | C-classification | SVM type for classification |
| scale | TRUE | Whether to scale features |
| probability | TRUE | Whether to compute class probabilities |
| coef0 | 0 | Independent term in kernel function (default) |

**Table S5. Key parameters of the KNN**

| Parameter Name | Value | Description |
| --- | --- | --- |
| k | 5 | Optimal number of nearest neighbors selected via 5-fold cross-validation. |
| prob | TRUE | Classification probabilities returned based on neighbor vote proportion. |
| use.all | TRUE | All equally distant neighbors are used when ties occur. |
| preprocessing | center, scale | Features were standardized using z-score normalization. |
| distance | Euclidean | Euclidean distance is used to compute similarity between samples. |

**Table S6. Key parameters of the LightGBM**

| Parameter Name | Value | Description |
| --- | --- | --- |
| learning_rate | 0.05 | Step size shrinkage is used to prevent overfitting and ensure better convergence. |
| num_leaves | 5 | Maximum number of leaves in one tree; controls model complexity. |
| max_depth | 3 | Maximum depth of a tree; limits the depth to avoid overfitting. |
| in_data_in_leaf | 10 | Minimum number of samples in one leaf node; prevents creating overly specific leaves. |
| feature_fraction | 0.8 | Fraction of features randomly selected for each boosting iteration. |
| nrounds | 300 | Total number of boosting iterations in the final training. |
| early_stopping_rounds | 30 | Early stopping is applied during CV to avoid overfitting. |
| feature_pre_filter | FALSE | Disabled automatic feature pre-filtering based on importance ranking. |
| min_data_in_bin | 1 | Minimum number of samples per histogram bin |
| min_gain_to_split | 0 | Minimal gain required to perform a split (default). |
| min_sum_hessian_in_leaf | 1 | Minimum sum of Hessian values in a leaf node |

**Table S7. Metabolic characteristics of three groups**

|  | Control(n=212) | False-negative(n=53) | True-positive(n=66) | P (three groups) | P (False-negative vs controls) | P (True-positive vs controls) |
| --- | --- | --- | --- | --- | --- | --- |
| Cit(µmol/L) | 13.59(11.80,16.89) | 25.50(19.82,30.03) | 97.06(66.45,204.44) | <0.001 | <0.001 | <0.001 |
| Phe(µmol/L) | 59.00(52.52,66.62) | 49.60(43.92,55.60) | 81.97(61.83,119.54) | <0.001 | <0.001 | <0.001 |
| Arg(µmol/L) | 5.33(2.93,8.61) | 10.58(4.98,16.06) | 17.69(10.57,33.04) | <0.001 | <0.001 | <0.001 |
| Orn(µmol/L) | 126.64(100.88,150.54) | 149.62(129.60,182.92) | 162.13(125.94,194.76) | <0.001 | <0.001 | <0.001 |
| Gly(µmol/L) | 535.49(453.84,634.52) | 390.39(341.44,478.79) | 352.38(279.82,469.92) | <0.001 | <0.001 | <0.001 |
| Pro(µmol/L) | 200.57(175.78,241.13) | 240.23(196.16,297.43) | 222.74(183.99,270.09) | <0.001 | <0.001 | <0.05 |
| SA(µmol/L) | 0.68(0.50,0.89) | 0.80(0.68,0.95) | 0.77(0.60,1.04) | <0.001 | <0.001 | <0.01 |
| C10:2(µmol/L) | 0.01(0.01,0.01) | 0.01(0.01,0.02) | 0.01(0.01,0.02) | <0.001 | <0.001 | <0.05 |

Normal range: Cit: 7.9-37µmol/L; Phe: 23.3-100µmol/L; Arg:2.54-50µmol/L; Orn: 52.09-323.22µmol/L; Gly:246.57-1283µmol/L; Pro: 97.2-401.5µmol/L; SA:0.16-2.58µmol/L; C10:2: 0.01-0.08µmol/L
